# SEPT12 phosphorylation results in loss of the septin ring/sperm annulus, defective sperm motility and poor male fertility

**DOI:** 10.1371/journal.pgen.1006631

**Published:** 2017-03-27

**Authors:** Yi-Ru Shen, Han-Yu Wang, Yung-Che Kuo, Shih-Chuan Shih, Chun-Hua Hsu, Yet-Ran Chen, Shang-Rung Wu, Chia-Yih Wang, Pao-Lin Kuo

**Affiliations:** 1 Department of Obstetrics and Gynecology, College of Medicine, National Cheng Kung University, Tainan, Taiwan; 2 Department of Biochemistry and Molecular Cell Biology, School of Medicine, College of Medicine, Taipei Medical University, Taipei, Taiwan; 3 Department of Biochemistry and Molecular Biology, National Cheng Kung University, Tainan, Taiwan; 4 Department of Agricultural Chemistry, National Taiwan University, Taipei, Taiwan; 5 Agricultural Biotechnology Research Center, Academia Sinica, Taipei, Taiwan; 6 Institute of Oral Medicine, College of Medicine, National Cheng Kung University, Tainan, Taiwan; 7 Department of Cell Biology and Anatomy, College of Medicine, National Cheng Kung University, Tainan, Taiwan; 8 The Institute of Basic Medical Sciences, College of Medicine, National Cheng Kung University, Tainan, Taiwan; University of Massachusetts Amherst, UNITED STATES

## Abstract

Septins are critical for numerous cellular processes through the formation of heteromeric filaments and rings indicating the importance of structural regulators in septin assembly. Several posttranslational modifications (PTMs) mediate the dynamics of septin filaments in yeast. However, little is known about the role of PTMs in regulating mammalian septin assembly, and the *in vivo* significance of PTMs on mammalian septin assembly and function remains unknown. Here, we showed that SEPT12 was phosphorylated on Ser198 using mass spectrometry, and we generated SEPT12 phosphomimetic knock-in (KI) mice to study its biological significance. The homozygous KI mice displayed poor male fertility due to deformed sperm with defective motility and loss of annulus, a septin-based ring structure. Immunohistochemistry of KI testicular sections suggested that SEPT12 phosphorylation inhibits septin ring assembly during annulus biogenesis. We also observed that SEPT12 was phosphorylated via PKA, and its phosphorylation interfered with SEPT12 polymerization into complexes and filaments. Collectively, our data indicate that SEPT12 phosphorylation inhibits SEPT12 filament formation, leading to loss of the sperm annulus/septin ring and poor male fertility. Thus, we provide the first *in vivo* genetic evidence characterizing importance of septin phosphorylation in the assembly, cellular function and physiological significance of septins.

## Author summary

Septin polymerization into filaments and rings are built for numerous cellular processes, and misregulation of septin filaments is implicated in several human diseases. Therefore, it is important to identify structural regulator of septin assembly. We showed that SEPT12 was phosphorylated on the Ser198 residue, and phosphomimetic SEPT12 KI mice display defective male fertility, impaired sperm motility and loss of septin-organized sperm annulus. Homozygous KI spermatids exhibited complete absence of septin ring assembly during annulus biogenesis. Additionally, SEPT12 phosphorylation through PKA interfered with SEPT12 polymerization into complexes and filaments. Thus, SEPT12 phosphorylation regulates SEPT12 polymerization that is involved in the septin ring assembly/sperm annulus establishment, the structural and mechanical support of sperm and robust male fertility. The present study provides novel insight into spermatogenetic regulation, highlights the importance of phosphorylation as structural regulator of septin assembly *in vivo*, and identifies the mechanism by which phosphorylation contributes to septin assembly.

## Introduction

Septins are highly conserved GTP-binding proteins in eukaryotes that polymerize into heteromers and further higher-order structures, such as filaments, rings and gauzes. These structures are required for a wide range of cellular processes, including cell cycle regulation, cytokinesis, spermiogenesis, ciliogenesis and neurogenesis [1–7]. Accordingly, misregulation of septin filaments is associated with diseases including cancer, male infertility and neurodegenerative disorders [8]. Given the critical roles of higher-order septin structures in diverse cellular processes, it is important to investigate the regulation of septin assembly.

Posttranslational modifications (PTMs) of yeast septins, such as sumoylation, phosphorylation and acetylation, modulate the dynamics of septin filament formation during the yeast cell cycle [9]. For example, the *Saccharomyces cerevisiae* septins are sumoylated during the cell cycle, and mutation of sumoylation sites interferes with the disassembly of the septin ring at a previous site of division [10]. In addition, the loss of PP2A-dependent dephosphorylation of yeast Shs1 results in failure to the remove septin ring at the bud neck at the completion of cytokinesis [11]. Interestingly, different phosphomimetic mutations in yeast Shs1 lead to no filament formation or distinct organizations of higher-order structures, such as rings and gauze-like structures [12]. However, the mechanism underlying the contribution of PTMs to the dynamics of higher-order structures remains unknown.

In mammalian cells, several studies have identified PTMs on septins, but little is known about the roles of PTMs in septin assembly. Previous findings have implicated the phosphorylation of SEPT3 by cGMP-dependent protein kinase I (PKG-I) in nerve terminals in presynaptic plasticity [13, 14]. SEPT4 is phosphorylated by GSK3, and during the epididymal transit of spermatozoa, inhibition of SEPT4 phosphorylation through Wnt/GSK3 signaling establishes the function of membrane diffusion barrier in the sperm tail [15]. SEPT5, which may be involved in synaptic vesicle transport, is ubiquitinated by E3 ligase Parkin for subsequent degradation [16]. However, whether these modifications mediate septin assembly or disassembly of higher-order structures remains largely unknown. More importantly, the *in vivo* significance of PTMs on septin assembly and function has not been explored.

The sperm annulus is a septin-organized fibrous ring in the sperm tail, and defects in the annulus have been associated with asthenoteratozoospermia and male infertility [17, 18]. Several septins, including SEPT 1, 2, 4, 6, 7, and 12, have been found to localize to the sperm annulus, and sterile men exhibiting abnormal sperm structure and motility display reduced septin signals[3, 5, 19, 20]. Additionally, *Sept4* null mice show reduced sperm motility, bent sperm tails and male infertility, and electron microscopy analysis of the sperm tail revealed the complete absence of an annular structure [3, 5]. These data suggest that septins are fundamental subunits for annular establishment and function. Notably, SEPT12 is specifically expressed in male germ cells and organizes as SEPT12-7-6-2 and SEPT12-7-6-4 filaments at the sperm annulus [6, 19]. A mutation of the SEPT12 GTP-binding site (SEPT12^D197N^) that abolishes SEPT12 polymerization into filaments leads to the absence of an annulus, abnormal structure and poor motility of spermatozoa in both infertile men and knock-in (KI) mice [6, 21]. These findings suggest that SEPT12 filament formation is required for septin ring/annulus establishment and proper sperm function. Taken together, these results indicate that sperm annulus/septin ring formation requires the polymerization of septin subunits into filaments and is involved in the normal function and structure of sperm and robust male fertility. Although the composition and importance of the sperm annulus have been investigated, the regulation of annular formation remains unknown.

In the present study, we showed that SEPT12 was phosphorylated at Ser198, and we generated KI mice harboring a phosphomimetic mutation to imitate constitutively phosphorylated SEPT12. Phosphomimetic SEPT12 KI mice displayed poor male fertility, abnormal sperm structure and motility, and loss of the annulus/septin ring. Moreover, we investigated how SEPT12 phosphorylation contributes to the absence of the annulus/septin ring and identified the regulatory kinase involved. In conclusion, we have established the first genetic model for characterizing the importance of mammalian septin phosphorylation *in vivo* and identified the mechanism underlying the role of phosphorylation in septin assembly.

## Results

### Phosphomimetic SEPT12 KI mice showed poor male fertility, abnormal sperm structure and weak motility

**Fig 1:**
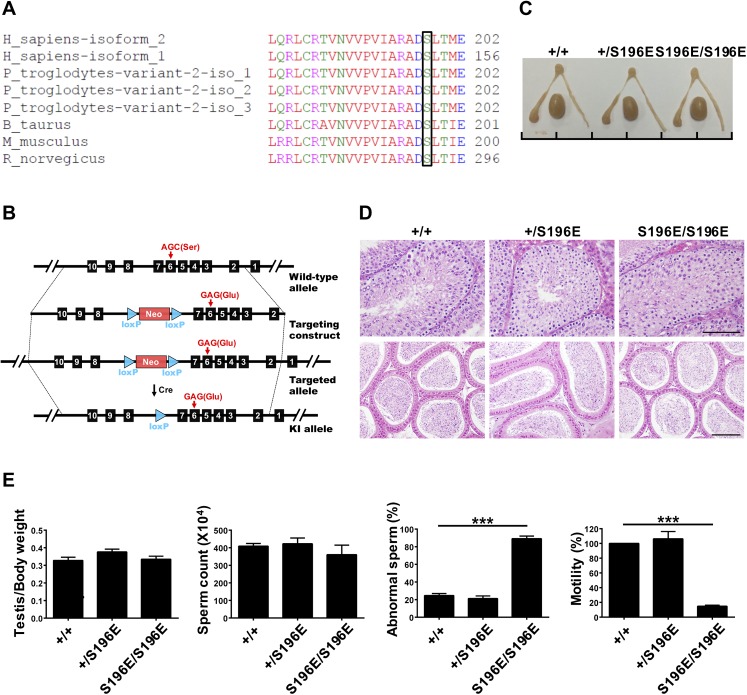
SEPT12^S196E/S196E^ mice displayed abnormal sperm morphology and motility. (A) Comparison of amino acid sequences flanking the Ser198 analogous sites of SEPT12 in various species using the ClustalW2 program at EMBL-EBI. The bracketed amino acid residues are analogous sites of Ser198. (B) Schematic illustration of the strategy for generating the SEPT12^S196E^ KI allele in embryonic stem cells. (C) Gross morphology of the testis and epididymis from WT and SEPT12 KI mice. Scale bar, 1 cm per unit. (D) Hematoxylin and eosin staining of testicular sections of stage XII tubule (top) and epididymal (bottom) sections from WT and SEPT12 KI mice. Scale bar, 100 μm. (E) Quantitative representation of testis/body weight, sperm counts, abnormal sperm morphology and sperm motility from WT and SEPT12 KI mice (Each genotype, N = 5). The sperm were isolated form the vas deferens. The data are represented as the means ± SEM. ***P<0.001.

The GFP-hSEPT12 protein was expressed in cells, and SEPT12 was immune-precipitated using the anti-GFP antibody followed by SDS-PAGE. The band corresponding to GFP-SEPT12 was excised, digested in-gel with trypsin and subjected for analysis by mass spectrometer. The MS/MS spectrum showed a phosphorylated SEPT12 peptide “196-ADSpLTMEER-204” indicating a phosphorylation site at Ser198 residue of SEPT12 (S1A Fig). The Ser198 residue is located in the SEPT12 GTP-binding domain and is highly conserved in mammals, as shown through multiple sequence alignment (Fig 1A).

To investigate the impact of SEPT12 phosphorylation *in vivo*, we established KI mice harboring a phosphomimetic mutation at the conserved Ser198 site to imitate constitutively phosphorylated SEPT12. KI mice carrying a mutation resulting in a change of the 196^th^ amino acid from serine to glutamate (SEPT12^S196E^) were generated, and the genotypes of the mice were determined through sequencing (Fig 1B and S1B Fig). The heterozygous KI males showed normal fertility (Table 1). However, the mating of homozygous KI males with distinct wild-type (WT) females for at least 4 months revealed a marked decrease in number of delivered litters and born pups compared with those of heterozygous males (Table 1). These data indicated SEPT12^S196E/S196E^ males displayed poor fertility. Analysis of the reproductive organs revealed no gross differences in testicular and epididymal morphology between WT and SEPT12 KI mice (Fig 1C). Additionally, histological analysis of these mice showed a similar germ cell population in the testis and densely packed tubules in the epididymis (Fig 1D). These findings are consistent with the absence of a significant difference in the testis/body weight ratio and sperm count (Fig 1E). However, a majority of spermatozoa from homozygous KI males exhibited abnormal structures and lacked motility (Fig 1E). Thus, SEPT12 homozygous KI males displayed poor fertility due to defective sperm morphology and motility, rather than abnormal reproductive organs.

**Table 1:** Male fertility of SEPT12^S196E^ heterozygous and homozygous mice.

	No. of males	No. of mating females	Mating duration (months)	No. of litters delivered	No. of pups born
SEPT12^+/S196E^	5	10	4	26	164
SEPT12^S196E/S196E^	5	10	4	1	3

No., number.

### Loss of the sperm annulus/septin ring in SEPT12^S196E/S196E^ mice

**Fig 2:**
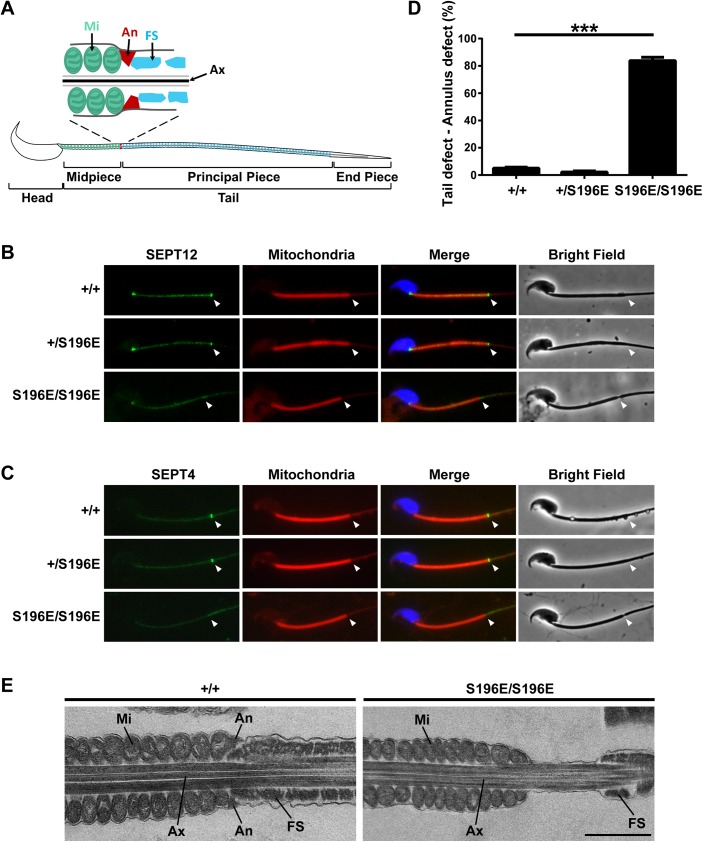
The annulus was absent in SEPT12^S196E/S196E^ spermatozoa. (A) Schematic representation of mouse spermatozoa. The annulus (An) is located between the mitochondria (Mi) in the midpiece and the fibrous sheath (FS) in the principal piece of the sperm tail. Ax, axoneme. (B, C) Immunofluorescence images of SEPT12 (B, green) or SEPT4 (C, green) from WT and SEPT12 KI spermatozoa. The middle piece was visualized through mitochondrial staining (Mito-Tracker, red). Nuclear staining (DAPI, blue) and bright-field images are also shown. Annular regions are indicated with arrowheads. (D) Quantitative representation of the sperm tail defect in the annular region from WT and SEPT12 KI mice (Each genotype, N = 5). The data are presented as the means ± SEM. ***P<0.001. (E) Electron microscopy analysis of WT and SEPT12 homozygous KI spermatozoa. In WT spermatozoa, the annulus (An) was observed between the mitochondria (Mi) and fibrous sheath (FS). In contrast, the annulus was completely lost in SEPT12 homozygous KI spermatozoa. The sperm were isolated form the epididymal cauda. Ax, axoneme. Scale bar: 1 μm.

Mature spermatozoa are comprised of head and tail which divided into a midpiece, principal piece and end piece (Fig 2A). The tail contains a central bundle of microtubules, the axoneme, enclosed by a mitochondrial sheath in the midpiece and a fibrous sheath in the principal piece. Between the mitochondrial and fibrous sheaths is the annulus, a septin ring [18]. Immunofluorescence analysis of WT spermatozoa showed SEPT12 and SEPT4 signals at the distal end of mitochondria, in the region of the annulus. However, these two signals were not detected in homozygous spermatozoa (Fig 2B and 2C). Statistical analysis showed that >80% of homozygous KI spermatozoa from epididymal cauda exhibited defects in the annular region, and these spermatozoa were either sunken or bent in the annular region observed by phase-contrast microscopy (Fig 2D and S2A Fig). Assaying sperm from epididymis reveals a significantly increase in hairpin-like tail bend during epididymal transit (S2B Fig). The phenotype of SEPT12 KI spermatozoa is similar with several mutant mice carrying defective sperm annulus, such as *Septin4*- and *Tat1*- and *Ccny1*-null mice [3, 5, 15, 22, 23]. These results indicate that phosphomimetic SEPT12 KI mice have a defective annulus devoid of essential septin subunits leading to sperm deformation.

To examine the sperm annulus structure in more detail, electron microscopy was performed. WT spermatozoa exhibited electron-dense wedge-shaped annulus located between the mitochondrial and fibrous sheaths (Fig 2E). However, the annulus was completely lost in phosphomimetic SEPT12 KI spermatozoa. Interestingly, the KI sperm displayed a greater distance and a sink between the mitochondrial sheath and the fibrous sheath. These results suggested that SEPT12 phosphorylation regulates the formation of the sperm annulus that is involved in the structural support of the sperm tail.

**Fig 3:**
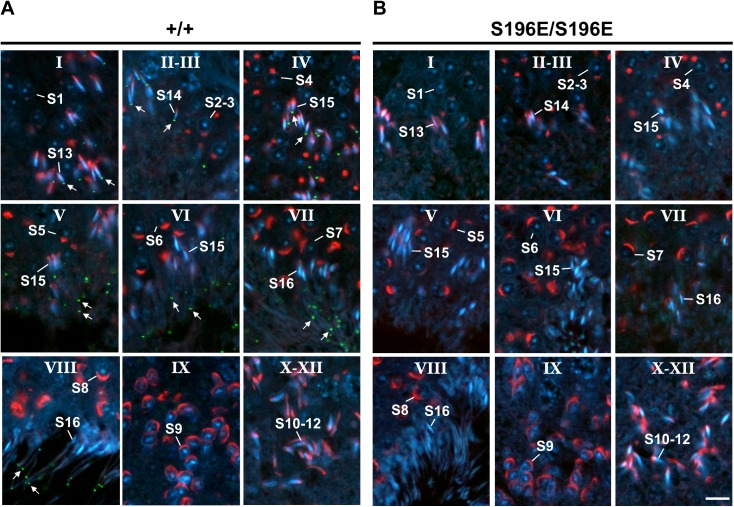
The annulus was completely absent in the seminiferous tubules of SEPT12^S196E/S196E^ mice during spermiogenesis. (A, B) Testicular sections of WT (A) and SEPT12^S196E/S196E^ (B) mice were stained to detect the annulus using an anti-SEPT4 antibody (green), the acrosomes using PNA lectin (red) and nuclei using DAPI (blue). Spermatogenetic stages were determined based on the morphology of the acrosome. The annulus is indicated with arrows. S1-16, step 1–16 spermatids. Scale bar: 10 μm.

To identify the annular defect in SEPT12 KI spermatozoa, we investigated the biogenesis of the annulus during spermatogenesis using SEPT4 as a representative annular marker [3, 5]. Spermatogenesis includes the mitotic phase, meiotic phase, and spermiogenesis and is divided into 12 stages in the seminiferous tubule, with each stage being characterized by a specific combination of germ-cell type [24]. Sperm annulus biogenesis only occurs in the spermatid during spermiogenesis, which can be subdivided into 16 steps in mice. Immunohistochemistry analysis of WT testicular sections showed that the annulus accumulates at the caudal pole of the nucleus in step 13 spermatids (Fig 3A; stage I) and retains its position until step 15 spermatids (Fig 3A; stage II–IV). Subsequently, the annulus continually moves down the sperm tail and arrives at the midpiece-principal piece junction in step 15–16 spermatids (Fig 3A; stage V-VIII). Strikingly, the annular signal was absent in all SEPT12 homozygous KI spermatids, indicating a lack of septin ring assembly at the beginning of annulus formation (Fig 3B). However, examination of SEPT4 expression revealed no significant difference between WT and SEPT12 KI spermatozoa (S3A Fig), and annular components including septin 2, 4, 6, 7 and 12 showed similar expression in WT and SEPT12 KI testis (S3B Fig). These indicate loss of sperm annulus in SEPT12 KI mice do not result from defective septin expressions. Collectively, these data suggest that SEPT12 phosphorylation interferes with the initial assembly of the septin ring, leading to a loss of sperm annulus formation.

### Mimetic phosphorylated Ser198 of SEPT12 disrupted SEPT12 filament formation through dissociation of SEPT12 from SEPT7-SEPT6-SEPT2 and SEPT7-SEPT6-SEPT4

To explore the molecular mechanism of SEPT12 phosphorylation in the assembly of higher-order structures, the effect of phosphomimetic SEPT12 on SEPT12 filament formation was examined in NT2/D1 cells, a human testicular carcinoma cell line.

**Fig 4:**
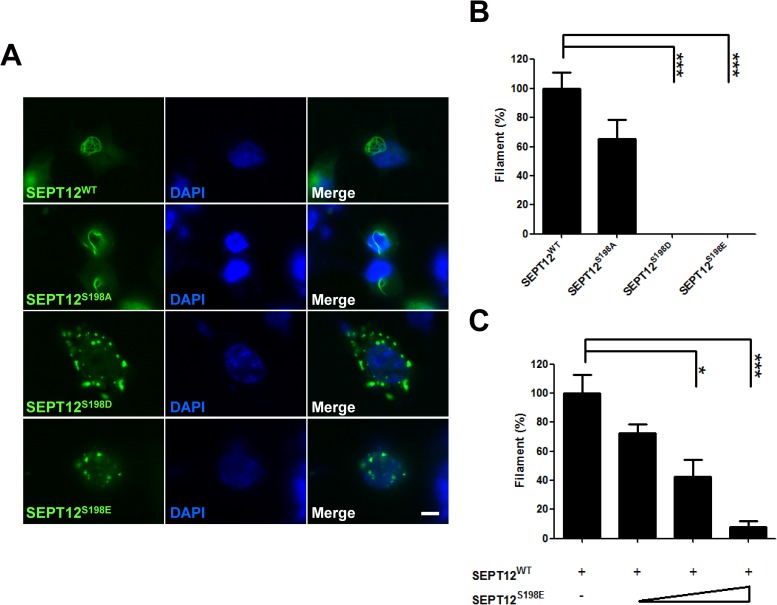
The expression patterns of mimetic phosphorylated Ser198 of SEPT12. (A, B) The GFP-SEPT12WT, GFP-SEPT12S198A, GFP-SEPT12S198D, and GFP-SEPT12S198E plasmids were transfected into NT2/D1 cells. (A) Different patterns of GFP-SEPT12 are shown. Scale bar, 10 μm. (B) The percentage of NT2/D1 cells with GFP filament fibers. (C) Effect of mimetic Ser198 phosphorylation of SEPT12 on SEPT12 filament formation. A total of 1 μg of the GFP-SEPT12WT plasmid was mixed with 0, 1, 2 or 4 μg of the GFP-SEPT12S198E plasmid and transfected into NT2/D1 cells. In B and C, the quantification bar was based on the observation of more than 100 cells for each experiment, and cells with GFP filament fibers were counted. The data are represented as the means ± SEM (n = 3). * P < 0.05, and *** P < 0.001.

The Ser198 residue of human SEPT12 was substituted with aspartate (S198D) or glutamate (S198E) to mimic the constitutively phosphorylated protein, or replaced with alanine (S198A) to imitate the unphosphorylated protein. GFP-SEPT12WT and S198A assembled into filamentous structures, but GFP-SEPT12S198D and S198E formed irregular aggregates in NT2/D1 cells (Fig 4A). Counting the cells with GFP-labeled filament fibers revealed that both SEPT12S198D and S198E resulted in no filament formation, indicating that Ser198 phosphorylation disrupts SEPT12 filament formation (Fig 4B). We next asked whether the mimetic phosphorylated Ser198 of SEPT12 could counteract filament formation by wild-type SEPT12. A constant amount of the GFP-SEPT12WT plasmid mixed with increasing doses of the phosphomimetic SEPT12S198E construct were transfected into cells, and the cells with filamentous structures were counted (Fig 4C). The results showed that quantity of SEPT12 filaments was gradually reduced, suggesting that elevated Ser198 phosphorylation might impair SEPT12 filament formation in a dose-dependent manner.

**Fig 5:**
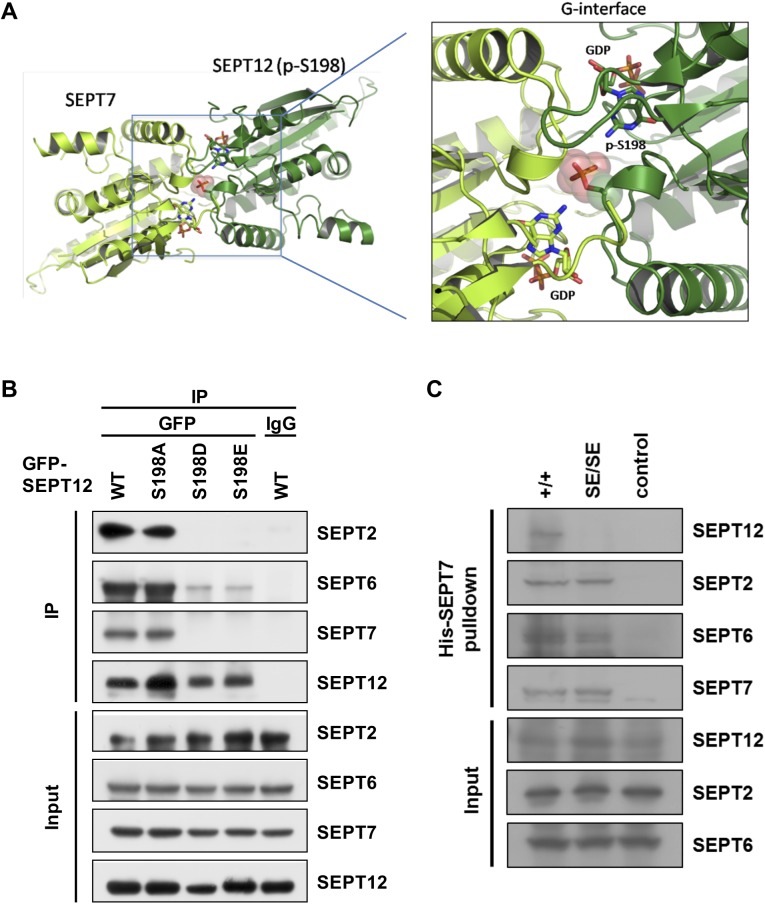
Mimetic phosphorylated Ser198 of SEPT12 disrupts SEPT12-7-6-2 complex. (A) The 3D-structure of the SEPT12-SEPT7 complex model shows that phosphorylated Ser198 (p-S198) of SEPT12 (shown in pink) is located at the dimeric G-interface. The structure of SEPT12 was built based on SEPT7 (PDB code: 3T5D) using the homology modeling method on the Swiss-model server (http://swissmodel.expasy.org/). (B) Myc-SEPT6 and FLAG-SEPT7 were co-transfected with various GFP-SEPT12 plasmids into NT2/D1 cells, and the cell lysates were immunoprecipitated using an anti-GFP antibody. The expression of SEPT2, 6, 7 and SEPT12 was detected using anti-SEPT2, anti-Myc, anti-FLAG and anti-GFP antibodies, respectively. (C) His-SEPT7 pull-down assay in WT and SEPT12 KI testis. His-SEPT7 recombinant protein was incubated with Ni-NTA beads followed by testicular lysate from WT and SEPT12^S196E/S196E^ mice. The pull-down protein was collected and subjected to western blotting for SEPT12, SEPT2 and SEPT6. The level of His-SEPT7 was detected using anti-His antibody. For the control group, WT testicular lysate was incubated with BSA instead of His-SEPT7.

The effect of SEPT12 S198 phosphorylation was further examined through 3D structure prediction using homology modeling revealing the phosphorylation of the SEPT12 Ser198 residue located at the SEPT12-SEPT7 interface (Fig 5A). It is known that SEPT12 polymerizes into SEPT12-7-6-2-2-6-7-12 and SEPT12-7-6-4-4-6-7-12 octamers, and these complexes further assemble into filaments through end-to-end associations [6]. Thus, we investigated whether Ser198 phosphorylation affects this assembly process, thereby disrupting SEPT12 filament formation. Immunoprecipitation analysis showed that SEPT12WT and S198A associated with SEPT2, SEPT6 and SEPT7, whereas the interaction between phosphomimetic SEPT12 (S198D and S198E) and these septins was profoundly or moderately perturbed (Fig 5B). These data suggested that Ser198 phosphorylation at the SEPT12-SEPT7 interface results in dissociation of SEPT12 from SEPT7, SEPT6 and SEPT2. Moreover, SEPT12 WT and S198A formed filamentous structures with SEPT7, SEPT6 and SEPT2, whereas phosphomimetic SEPT12 (S198D and S198E) showed no filament formation and disassociated from these septins. Importantly, SEPT7, SEPT6 and SEPT2 remained co-localized with each other, suggesting dissociation of phosphomimetic SEPT12 from the entire SEPT7-6-2-2-6-7 complex (S4 Fig). Collectively, these results indicated that SEPT12 Ser198 phosphorylation disrupts filament formation by interfering with the SEPT12-SEPT7 interaction, thereby inhibiting the association between SEPT12 and the SEPT7-6-2-2-6-7 complex. Similarly, phosphomimetic SEPT12 (S198D and S198E) inhibited the interaction of SEPT12 with SEPT4, SEPT6 and SEPT7 and resulted in dissociation of SEPT12 from the SEPT7-6-4 complex (S5A and S5B Fig).

In support of these findings in NT2/D1 cells, pull-down assay showed that His-tag SEPT7 recombinant protein interacted with SEPT12, SEPT2 and SEPT6 in wild-type testis. In SEPT12 KI testis, however, the association between phosphomimetic SEPT12 and His-SEPT7 was abolished, but SEPT7 still interacted with SEPT2 and SEPT6 (Fig 5C). Additionally, co-immunoprecipitation of SEPT2 indicated that SEPT2 still interacted with SEPT6 and SEPT7 in SEPT12 KI testis (S6 Fig). Thus, SEPT12 phosphorylation disrupts association between SEPT12 and SEPT7, but pre-complex SEPT7-6-2 still assemble *in vivo*. Together, these results suggest that SEPT12 Ser198 phosphorylation disrupts sperm annuls formation by abolishing assembly of SEPT12 with pre-complex SEPT7-6-2.

In contrast, phosphomimetic SEPT12 (S198D and S198E) did not affect the SEPT12-SEPT12 interaction, which is required for the association between octamers (S7A Fig). Additionally, phosphomimetic SEPT12 (S198D and S198E) remained co-localized with SEPT12WT (S7B Fig). In conclusion, SEPT12 phosphorylation destroys filament formation by disrupting the intra-complex association, rather than the inter-complex association.

### PKA phosphorylated the SEPT12 Ser198 residue to modulate SEPT12 filament formation

**Fig 6:**
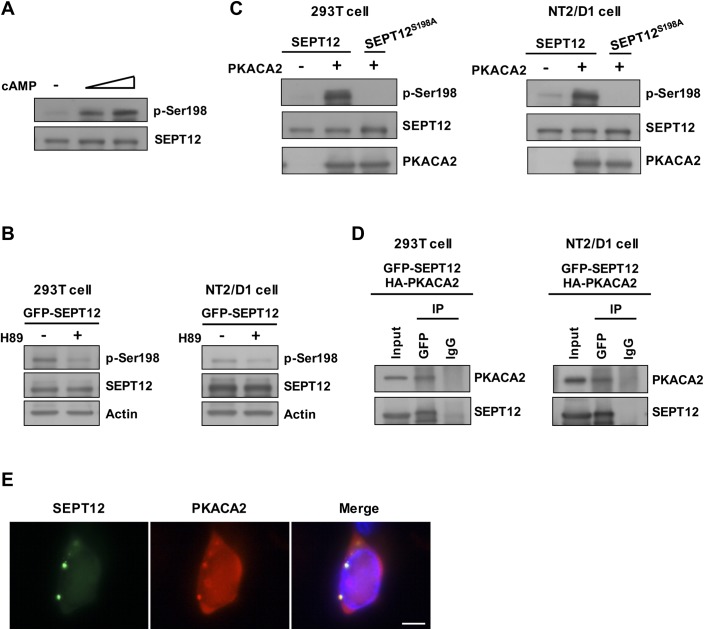
The SEPT12 Ser198 residue was phosphorylated by PKA. (A) The Ser198 phosphorylation of SEPT12 (p-Ser198) was gradually elevated through increasing the dosage of cAMP treatment. GFP-SEPT12 was overexpressed in 293T cells, followed by treatment with 0, 1, and 5 mM cAMP, and the expression of phosphorylated Ser198 and SEPT12 was detected using anti-phospho-Ser198 and anti-GFP antibodies, respectively. (B) GFP-SEPT12 was overexpressed in 293T and NT2/D1 cells, followed by treatment with 50 μM H89, and the expression of phosphorylated Ser198, SEPT12 and actin was detected using anti-phospho-Ser198, anti-GFP and anti-Actin antibodies, respectively. (C) GFP-SEPT12 and GFP-SEPT12S198A, with or without HA-PKACA2, were transfected into 293T cells and NT2/D1 cells as indicated, and the expression of phosphorylated Ser198, SEPT12 and PKACA2 was detected using anti-phospho-Ser198, anti-GFP and anti-HA antibodies, respectively. (D) Co-immunoprecipitation of GFP-SEPT12 with HA-PKACA2. 293T Cells or NT2/D1 cells were transfected with GFP-SEPT12 and HA-PKACA2, and the lysates were immunoprecipitated using an anti-GFP antibody. Immunoblotting was performed using anti-GFP and anti-HA antibodies. (E) SEPT12 and PKACA2 showed partial co-localization. GFP-SEPT12 and HA-PKACA2 were co-expressed in NT2/D1 cells, and immunofluorescence was detected using an anti-HA antibody. Scale bar, 10 μm.

Inspection of the SEPT12 Ser198 residue and its flanking sequence identified a consensus target motif of protein kinase A (PKA), [R/K]-X-X-[pS/T] (Fig 1A) [25]. PKA is a heterotetramer comprising two regulatory and two catalytic subunits; upon cAMP stimulation, the catalytic subunits dissociate from the regulatory subunits, thereby activating downstream cascades. We examined whether PKA is responsible for SEPT12 Ser198 phosphorylation using a phospho-Ser198 antibody that specifically recognizes SEPT12 phosphorylation at the Ser198 residue (S8 Fig). Cells were treated with an increasing dose of cAMP followed by western blotting revealing that SEPT12 phosphorylation at the Ser198 residue (p-Ser198) was elevated in a dose-dependent manner (Fig 6A). In addition, inhibition of PKA activity by H89 reduced p-Ser198 signal in both 293T and NT2/D1 cells (Fig 6B). These findings suggested a positive correlation between PKA activation and Ser198 phosphorylation status. The catalytic subunit alpha 2 of PKA (PKACA2) is exclusively expressed in the testis and is the predominant catalytic form of PKA during spermiogenesis, specifically, in the stage of annular biogenesis [18, 19, 26]. Thus, we examined whether PKACA2 promotes SEP12 phosphorylation. Overexpression of PKACA2 markedly increased the p-Ser198 signal of SEPT12WT, but not that of S198A, in both the 293T and NT2/D1 cell lines, indicating that PKACA2 could facilitate SEPT12 phosphorylation at the Ser198 residue (Fig 6C). Moreover, immunoprecipitation and immunofluorescence analyses showed SEPT12 co-precipitated and co-localized with PKACA2, demonstrating that SEPT12 physically interacted with PKACA2 (Fig 6D and 6E). Taking these findings together, we concluded that PKACA2 associated with SPET12 and phosphorylated its Ser198 residue. Notably, homology alignment among human septins revealed a consensus PKA target sequence at the Ser198 analog site in a majority of human septins, except for SEPT1, SEPT5 and SEPT6 (S9 Fig). Thus, it is likely that a majority of human septins serves as downstream targets of PKA.

**Fig 7:**
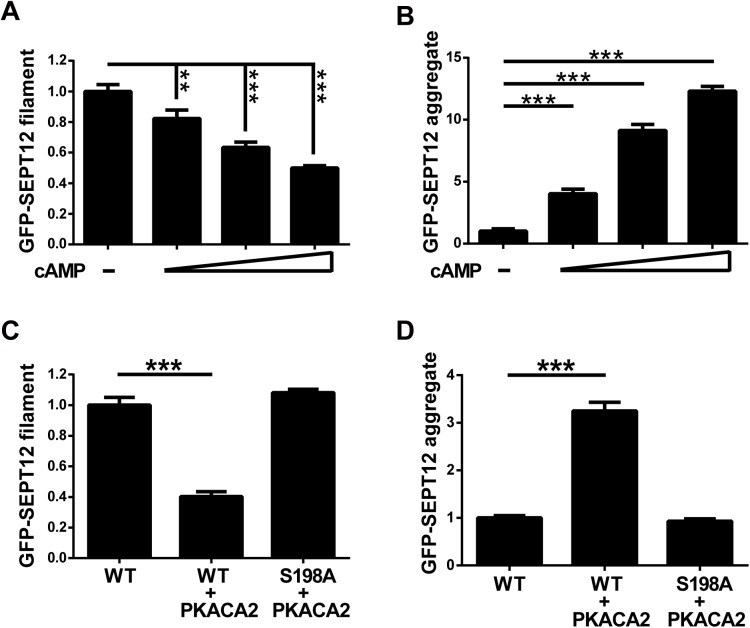
PKA disrupts SEPT12 filament formation via Ser198. (A, B) GFP-SEPT12 filaments were gradually decreased, and the aggregates were gradually increased in a cAMP dose-dependent manner. GFP-SEPT12 was overexpressed in 293T cells with 0, 1, 5, and 10 mM cAMP treatment, and cells with GFP-filament fibers (A) and GFP aggregates (B) were counted. (C, D) PKACA2 regulates SEPT12-organized structures through Ser198. GFP-SEPT12 or GFP-SEPT12S198A with or without HA-PKACA2 was overexpressed in 293T cells, and cells with GFP-filament fibers (C) and GFP aggregates (D) were counted. Each quantification bar was based on the observation of more than 500 cells. The data are represented as the means ± SEM (n = 3). ** P < 0.01, and *** P < 0.001.

We further examined the effect of PKA-dependent Ser198 phosphorylation on SEPT12-organized structures. Cells expressing GFP-SEPT12 were treated with cAMP, and the cells with GFP-labeled filaments or aggregates were subsequently counted. Elevating the dose of cAMP treatment gradually reduced the number of SEPT12 filaments and increased SEPT12 aggregates, suggesting that PKA activation determines the formation of SEPT12-organized structures (Fig 7A and 7B). Consistent with these findings, overexpressed PKACA2 decreased the filament formation of SEPT12WT, but not that of unphosphorylated SEPT12S198A in 293T cells (Fig 7C). In contrast, PKACA2 enhanced the aggregate accumulation of SEPT12WT, but not that of S198A (Fig 7D). Similar results were observed in NT2/D1 cells, in which PKACA2 altered SPET12WT-mediated, but not S198A-mediated structures (S10 Fig). These data indicated PKACA2 disrupts SEPT12 filament formation and promotes SEPT12 aggregate accumulation through the Ser198 residue. These findings correlated with the mimetic phosphorylated Ser198 of SEPT12 (SEPT12S198D and S198E) formed aggregates, but not filamentous structures (Fig 4A). In conclusion, PKA-dependent Ser198 phosphorylation impairs SEPT12 filament formation.

## Discussion

**Fig 8:**
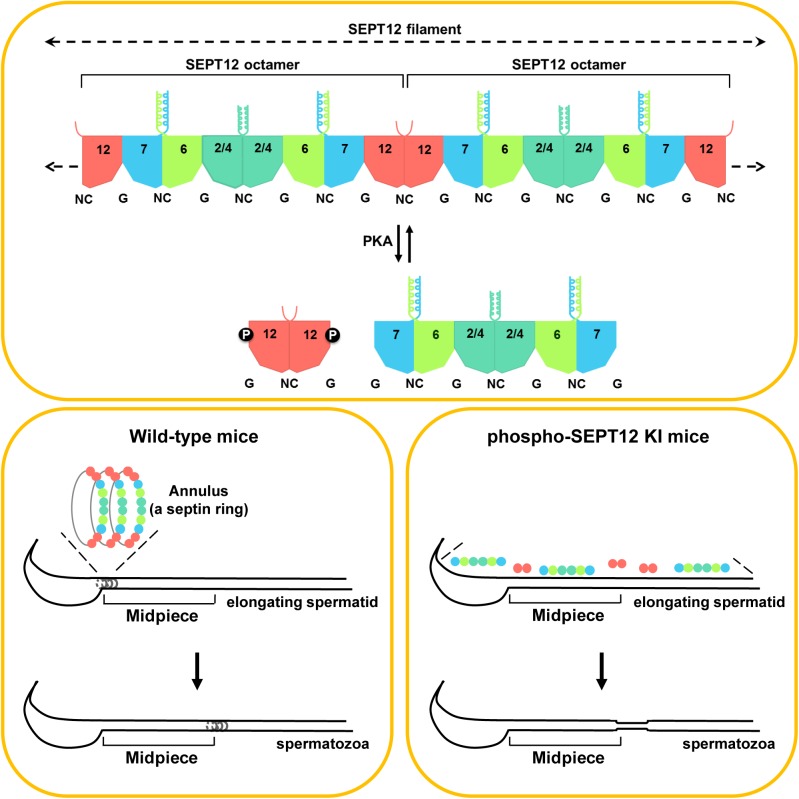
Working models for SEPT12 phosphorylation during sperm annulus biogenesis. SEPT12 filament is comprised of SEPT12-7-6-2-2-6-7-12 and SEPT12-7-6-4-4-6-7-12 octamers. Phosphorylation on SEPT12 Ser198 through PKA dissociates SEPT12 from the SEPT7-6-2 and SEPT7-6-4 complexes but does not disrupt the SEPT12-SEPT12 interaction. In WT mice, sperm annulus is assembled by SEPT12 filament at sperm neck and migrated along axoneme toward mid-principal piece junction. Disorganization of SEPT12 filament in phospho-SEPT12 KI mice fails to establishment of sperm annulus and lead to sperm deformation. The dotted arrows indicate the elongation of the filament with the addition of the SEPT octamers. NC, the N- and C-termini of the septin proteins. G, the GTP-binding domain of septins.

Here, we identified a phosphorylation site in SEPT12 and investigated its biological function during spermatogenesis (Fig 8). Sperm annulus, a septin ring, consists of SEPT12 filament, which is polymerized by end-to-end association of SEPT12-7-6-2-2-6-7-12 and SEPT12-7-6-4-4-6-7-12 octamers [6]. Upon phosphorylation through PKA at Ser198 residue, phospho-SEPT12 dissociates from SEPT7-6-2 and SEPT7-6-4 complexes, thereby disrupting SEPT12 filament formation. In wild-type mice, sperm annulus is assembled at sperm neck in elongating spermatid and subsequently migrates along axoneme to the mid-principal piece junction in spermatozoa. In phospho-SEPT12 knock-in mice, disorganized septin complexes are unable to polymerize into SEPT12 filament resulting in loss of sperm annulus, and leading to deformed sperm with weak motility and poor male fertility. In the present study, we provide the first *in vivo* evidence indicating phosphorylation as a structural determinant for septin assembly into higher-order structures, and the importance of phosphorylation in septin-mediated cellular and physiological function.

SEPT12 KI mice display defective sperm annulus devoid of SEPT4 and SEPT12 subunits (Fig 2B and 2C), is it possible that these KI mice could function as SEPT4 and/or SEPT12 KO mice? Although SEPT12 KI mice share several features with SEPT4-null mice, SEPT12 KI mice do not have all defect of SEPT4-null mice, for example, mitochondrial defects [5]. In addition, the phenotype of SEPT12 KI mice is less severe than SEPT12 knock-out chimera mice, which display decrease in sperm count, defects in several sperm compartments and maturation arrest of germ cells at the spermatid stage [19]. Moreover, similar septin expressions including SEPT2, 4, 6, 7 and 12 between WT and SEPT12 KI testis indicate the absence of sperm annulus do not result from the loss of septin expression (S3B Fig). We conclude that phosphomimetic SEPT12 KI mice appears to be a mouse model with presence of septin subunits but loss of septin-septin interaction and septin filament assembly.

Electron microscopy analysis of the sperm annulus revealed an electron-dense ring structure between the midpiece and principal piece of all mammalian spermatozoa [27]. High-magnification images showed that the annulus/ring structure consisted of highly packed filamentous structures. Consistent with the observed ultrastructure of the annulus, SEPT1, 2, 4, 6, 7 and 12 have been shown to localize at the sperm annulus, and septins are known to polymerize into filaments and bundles of filaments that can further form rings [3, 5, 6, 28]. These results suggest that the architecture of the annulus/ring comprises multiple layers of septin filaments. Strikingly, spermatozoa carrying a phosphomimetic mutation in SEPT12 displayed a complete loss of the annulus, implying that a single phosphorylation site in SEPT12 controls the formation of multiple septin filamentous layers, a ring structure. Therefore, the present study highlights the importance of PTMs in the assembly of higher-order septin structures *in vivo*.

Morphological description of the sperm annulus was performed decades ago, and studies concerning its composition and function are accumulating. Three functions of the annulus/septin ring of sperm have been demonstrated: (1) structural and mechanical support; (2) acting as a diffusion barrier to confine proteins to a specific membrane domain; and (3) establishment of the mitochondrial distribution [3, 5, 6, 29, 30]. In the present study, phosphomimetic SEPT12 KI spermatozoa with no annulus were found to show malformation and lack of motility, consistent with the known annulus function (Figs 1E and 2). Interestingly, the KI spermatozoa exhibited a greater distance between the mitochondrial and fibrous sheaths, causing a sink and hairpin-like bend in the tail. We proposed that the annulus/septin ring might serve as an adhesive connecting these two compartments protecting sperm from deformation. The role of the annulus in determining the mitochondrial distribution is controversial. In the midpiece of SEPT4-null spermatozoa, which have no annulus, the overall mitochondrial alignment is established, although the mitochondrial architecture is defective in terms of the cristae and size [5]. Here, we observed that SEPT12 KI spermatozoa exhibit normal mitochondrial morphology and distribution in the midpiece (Fig 2E). These indicate that mitochondrial defect is a SEPT4-null-specific phenotype, and the annulus is not required for establishing the mitochondrial distribution. In addition to providing insight into annular function, the present study shows that phosphomimetic mutation in SEPT12 disrupts annulus formation in KI sperm. These findings further suggest that SEPT12 phosphorylation serves as a structural determinant controlling sperm annular establishment.

During sperm transit in the epididymis, annulus could be a fence that close to act as membrane diffusion barrier confining proteins to specific region, or open as one-way gate for passage of proteins in one direction [29]. For example, basigin is restricted in the principal piece by annulus in caput sperm; however, it passes through the annulus and relocates to midpiece in cauda sperm [29, 31]. Koch et al. showed that in *Ccny1*-null sperm, which is deficient in Wnt signaling, SEPT4 phosphorylation through GSK3 leads to loss of high-molecular-weight SEPT4 complexes and impair barrier function of sperm annulus [15]. These results suggest that septin phosphorylation determines barrier function through affecting annulus structure. In the present study, we further demonstrate that SEPT12 phosphorylation interferes with septin-sepitn interaction leading to disrupt filament assembly of annulus structure. Collectively, we speculate that during epididymal transit of spermatozoa, septin phosphorylation status regulates septin assembly and disassembly leading to affect state of annulus fence (diffusion barrier and open gate) for protein restriction or translocation.

Higher-order septin structures, including filaments, rings and gauzes, are built for various cellular functions in organisms ranging from yeast to humans. However, the molecular mechanisms underlying the regulation of higher-order septin structures remain largely unknown. According to structural studies, all septins comprise highly conserved G domains and divergent N- and C-termini [28]. Electron microscopy analyses of the septin complexes of *C*. *elegans*, *S*. *cerevisiae* and *H*. *sapiens* indicate that septins self-polymerize into core complexes with mirror symmetry and alternative G- and NC-interfaces [32–34]. Additionally, septin complexes join end-to-end to form filaments under a low ionic strength *in vitro* or by membrane-directed annealing in yeast [32–35]. These results imply identical assembly fashion to all septin complexes and higher-order septin structures. Thus, given the shared domain structure and mode of assembly, the basic mechanism in regulating dynamics of septin complexes and/or higher-order structures might be common to septins. In the present study, we observed that SEPT12 Ser198 lies in the conserved G domain, and Ser198 phosphorylation abolishes the SEPT12-SEPT7 association and thereby disrupts SEPT12 filaments. Thus, it is likely a general phenomenon that septin PTMs in a conserved G domain regulate assembly of complex and higher-order structures through the direct modulation of SEPT-SEPT association.

We observed that the SEPT12 Ser198 residue was phosphorylated by PKA to impair SEPT12 filament formation and promote the accumulation of SEPT12 aggregates. Notably, the PKA target motif is found not only in SEPT12 but also in other human SEPTs, suggesting that these human septins are subject to PKA phosphorylation and might undergo changes in organized structures (S9 Fig). Thus, it is likely that PKA enables a majority of human septins to achieve structural diversity associated with different functional properties.

In conclusion, the results of the present study provide *in vivo* physiological evidence of the PTM importance in the assembly, cellular functions and physiological impact of mammalian septins. In addition, we identified the mechanism by which PTMs contribute to septin assembly. These findings highlight the involvement of mammalian septin PTMs in higher-order structure assembly and provide insight into the regulation of septin assembly.

## Materials and methods

### Mass spectrometry

The GFP-hSEPT12 plasmid was transfect into the 293T cells for 40 hours. SEPT12 was immune-precipitated using the anti-GFP antibody followed by SDS-PAGE and Coomassie Blue analysis. The band corresponding to GFP-SEPT12 (~72 kDa) was excised, digested in-gel with trypsin and subjected for analysis by mass spectrometer. Nanoflow liquid chromatography and tandem mass spectrometry (LC-MS/MS) was performed by coupling a linear ion trap mass spectrometer (LTQ Velos Pro, Thermo Fisher Scientific) to a nanoflow LC system (nanoACQUITY UPLC, Waters) using a tunnel frit trap column [36] packed (180 μm × 20 mm) with 5 um Symmetry C18 beads (Waters) and an analytical column (BEH130 C18, 1.7 um, 75 um × 250 mm, Waters). An acetonitrile/water gradient of 12–80% for 22 min was used for analysis of tryptic phosphopeptides. For MS analysis, up to ten ion-trap MS/MS spectra were acquired per data-dependent cycle from MS scan range 400 to 1600 m/z with the ion charge 2+, 3+, and 4+. The MS/MS spectra were converted into a peak list file and searched against the human International Protein Index database (version 3.61) using the MASCOT search algorithm (version2.3.0; Matrix Science). The mass tolerance in the database search for peptide MS and MS/MS were ±0.5 and ±1.5 Da, respectively. Methionine oxidation and phosphorylation on serine, threonine, and tyrosine residuals were set as variable modifications; cysteine methylthiolation was set as fix modification. The Mascot Delta (MD) score was obtained from the Mascot search result files by calculation the best and second best Mascot ion scores for the correct and alternative phosphorylation site localizations on an otherwise identical peptide sequence. The false position rate (FPR) of phosphorylation site determination was lower than 0.01 by using MD score higher than 19 which were published in 2011[37].

### Generation of SEPT12S196E KI mice

The Institutional Review Boards of National Cheng Kung University Medical Centre and National Taiwan University Medical Center approved all animal procedures. The entire Septin12 genomic fragment in the bMQ251k06 BAC clone was obtained from the BACPAC Resource Center, and the fragment containing exon 2 to exon 10 was cloned into the pL253 targeting vector (Fig 1B). A targeting construct with a serine-to-glutamate (S196E) substitution was introduced into exon 6 through site-directed mutagenesis, and a loxP-neo-loxP cassette was inserted between exon 7 and exon 8 of Septin12. The targeting construct was linearized and electroporated into mouse embryonic stem cells to replace the wild-type allele of Septin12. Cell clones with the targeted allele were selected through Southern blot analysis and transfected with the Cre recombinase-expressing plasmid to remove the cassette, resulting in the KI allele. Cell lines with the KI allele were confirmed through genotyping and injected into C57BL/6J blastocysts to generate chimeric mice. Male chimeric mice were mated with wild-type females to transmit the KI allele to their offspring. The heterozygous KI mice were bred to yield homozygous KI mice. The following primers were used for the genotyping of WT and KI mice: forward—5’-TTTCTTCCCTCACTCATCCAC-3’, and reverse—5’-TCTACAGCATCTTACCCGAATC-3’

### DNA constructs, cell culture, and transfection

The generation of all septins constructs and its mutant constructs was previously described [6]. NT2/D1 and 293T cells were cultured in Dulbecco’s minimal essential medium (DMEM) supplemented with 10% fetal bovine serum (FBS) and 1% antibiotics. For transient transfection, NT2/D1 and 293T cells were transfected using Lipofectamine 2000 (Invitrogen) according to the manufacturer’s instructions.

### Immunofluorescence staining and immunohistology

For immunofluorescence analysis, mouse spermatozoa and NT2/D1 cells were fixed with 4% paraformaldehyde in phosphate-buffered saline (PBS), permeabilized with 0.1% triton X-100 in PBS, and blocked with the antibody diluent (Dako). Spermatozoa and cells were subsequently incubated with an anti-SEPT2 (1:100, Proteintech), anti-SEPT4 (1:100, Santa Cruz Biotechnology), anti-SEPT12 (1:100; Abnova), anti-FLAG (1:200, Sigma-Aldrich), anti-Myc (1:200, Genetex) or anti-HA (1:200, Covance) antibody at 4°C overnight, followed by washing three times and Alexa Fluor 350-, Alexa Fluor 488-, Alexa Fluor 568- or Alexa Fluor 660-labeled antibody staining (1:200; Molecular Probes). Nuclei were counterstained with 4,6-diamidino-2-phenylindole (DAPI). For immunohistology, the testis and epididymis were fixed in Bouin’s solution overnight and embedded in paraffin blocks. The blocks were sliced into 5-μm thin sections and applied to glass slides. The sections were deparaffinized, rehydrated and stained with hematoxylin/eosin or incubated with the anti-SEPT4 (1:100) antibody and lectin peanut agglutinin (PNA)-conjugated with Alexa Fluor.

### Immunoprecipitation assay and western blot analysis

For immunoprecipitation analysis, 2 μg of an anti-GFP or anti-FLAG antibody was incubated with Dynabeads protein G (Thermo Fisher Scientific) at room temperature for 15 min on a rotator, and the cell lysates were immunoprecipitated with the bead-antibody complex at 4°C overnight. The beads were collected after brief centrifugation and washed three times with wash buffer (100 mM Tris, 150 mM NaCl, 2 mM EDTA, 0.5% Tween-20, and 0.01% NP-40). The precipitates were resuspended in SDS-sample buffer and denatured at 95°C for 10 min. For western blotting, the proteins were separated through sodium dodecyl sulfate-polyacrylamide gel electrophoresis (SDS-PAGE) and blotted onto PVDF membranes (Millipore). The blots were then incubated with an anti-SEPT2 (1:1000), anti-SEPT4 (1:250, sigma), anti-SEPT6 (1:1000, Santa Cruz Biotechnology), anti-SEPT7 (1:1000, Santa Cruz Biotechnology), anti-His (1:10000, Abcam), anti-GFP (1:4000), anti-FLAG (1:1000), anti-Myc (1:5000), anti-HA (1:5000) or anti-phospho-Ser198 (1:1000) antibody. Anti-phospho-Ser198 was purchased from Kelowna International Scientific, Inc. and was generated through immunizing rabbits with a phosphorylated peptide (RADpSLTMEEREA, amino acids 195 to 206 in NP_653206.2) of SEPT12.

### His pull-down assay

His-tagged SEPT7 recombinant protein was purchased form MyBiosource and 5 μg His-SEPT7 pre-incubated with 30 μl Ni-NTA beads (Qiagen) under 5mM imidazole for an hour at 4°C. Testicular lysate were then incubated with His-SEPT7-beads complexes under 5mM imidazole overnight at 4°C. The mixtures were washed three times and subjected to western blot analysis.

### Transmission electron microscopy

A total of 10^6^ mouse spermatozoa were collected from the cauda epididymis and pre-fixed with 2.5% glutaraldehyde at 4°C overnight. The prefixed spermatozoa were post-fixed in a 1% OsO4 solution at room temperature for 1 h, washed three times with ddH_2_O, dehydrated using ethanol and propylene oxide, embedded in Epon at room temperature, and polymerized in an oven at 55°C for 1 day. Finally, 80 nm thin sections were collected on grids, stained with lead citrate and uranyl acetate and examined through transmission electron microscopy (JEM-1400, JEOL).

### ClustalW multiple sequence alignment

The human SEPT12 orthologous proteins in various species were aligned using the ClustalW2 program provided by EMBL-EBI (http://www.ebi.ac.uk/). The accession numbers for SEPT12 proteins of different species were as follows: Homo sapiens (isoform 1: NP_001147930.1; isoform 2: NP_653206.2), Pan troglodytes (isoform 1: XP_001169473.1; isoform 2: XP_001169539.1; isoform 3: XP_001169556.1), Bos Taurus (NP_001091612.1), Mus musculus (NP_081945.1) and Rat tusnorvegicus (XP_343860.3). The accession numbers for SEPTIN proteins of Homo sapiens were as follows: SEPT1 (NP_443070.1), SEPT2 (NP_001008491.1), SEPT3 (isoform A: NP_663786.2; isoform B: NP_061979.3), SEPT4 (isoform 1: NP_004565.1; isoform 2: NP_536340.1; isoform 3: NP_536341.1), SEPT5 (NP_002679.2), SEPT6 (isoform A: NP_665798.1; isoform B: NP_055944.2; isoform D: NP_665801.1), SEPT7 (isoform 1: NP_001779.3; isoform 2: NP_001011553.2), SEPT8 (isoform A: NP_001092281.1; isoform B: NP_055961.1; isoform C: NP_001092282.1; isoform D: NP_001092283.1), SEPT9 (isoform A: NP_001106963.1; isoform B: NP_001106965.1; isoform C: NP_006631.2; isoform D: NP_001106967.1; isoform E: NP_001106964.1; isoform F: NP_001106968.1), SEPT10 (isoform 1: NP_653311.1; isoform 2: NP_848699.1), SEPT11 (NP_060713.1) and SEPT14 (NP_997249.2). The above information was adopted from the NCBI database (http://www.ncbi.nlm.nih.gov/).

### Statistical analysis

All data are presented as the means ± SEM. Statistical differences were analyzed through one-way analysis of variance (ANOVA), combined with the Tukey’s Multiple Comparison Test for posterior comparisons. P-values were considered significant at *P< 0.05; **P< 0.01; ***P<0.001.

## Supporting information

S1 Fig Detection of human SEPT12 PTM through mass spectrometry and identification of mouse genotypes. (A) Detection of human SEPT12 PTM through mass spectrometry. The MS/MS spectrum of the SEPT12 Ser198 phosphorylated peptide “196-ADSpLTMEER-204”; the y- and b-fragments for confirming Ser198 phosphorylation site are annotated. (B) Mouse genotypes were determined through DNA sequencing. Electropherograms show the genomic DNA sequence of wild-type, heterozygous and homozygous SEPT12 KI mice. Mutation from serine (Ser) to glutamate (Glu) was observed in one allele of heterozygous KI mice and in two alleles of homozygous KI mice.
(PDF)

S2 Fig SEPT12^S196E/S196E^ spermatozoa displayed tail bend at annulus region and increased hairpin-like structure during epididymal transit. (A) SEPT12^S196E/S196E^ sperm tails have various bending configuration. Spermatozoa were harvested form WT and SEPT12^S196E/S196E^ cauda followed by phase-contrast microscopy analysis. The bright-field images were shown and annular regions are indicated with arrowheads. (B) Quantitative representation of the hairpin-like bend of WT and SEPT12 KI spermatozoa from epididymal caput, corpus and cauda (Each genotype, N = 4). The data are presented as the means ± SEM. ***P<0.001.
(PDF)

S3 Fig Detection of septin expressions in spermatozoa and testis of wild-type and SEPT12^S196E/S196E^ KI mice. (A) SEPT4 expression in WT and SEPT12 KI spermatozoa from epididymal cauda. (B) The expression of septins including SEPT2, 4, 6, 7 and 12 in WT and SEPT12 KI testis.
(PDF)

S4 Fig Mimetic phosphorylated SEPT12 disrupted filament formation of SEPT12 with SEPT7-6-2. NT2/D1 cells were co-transfected with various plasmids, shown on the left. Immunofluorescence staining revealed the subcellular patterns of GFP-SEPT12, FLAG-SEPT7, Myc-SEPT6 and endogenous SEPT2 in the cells expressing wild-type or mutant SEPT12. Scale bar, 10 μm.
(PDF)

S5 Fig Mimetic phosphorylated Ser198 of SEPT12 disrupts SEPT12-7-6-4 complex and filament formation. (A) Co-transfection of HA-SEPT4, Myc-SEPT6 and FLAG-SEPT7 with various GFP-SEPT12 plasmids into NT2/D1 cells; lysates were immunoprecipitated using an anti-GFP antibody. The expression of SEPT4, 6, 7 and SEPT12 was detected using anti-HA, anti-Myc, anti-FLAG and anti-GFP antibodies, respectively. (B) NT2/D1 cells were co-transfected with various plasmids, as shown on the left. Immunofluorescence staining showed the subcellular patterns of GFP-SEPT12, FLAG-SEPT7, Myc-SEPT6 and HA-SEPT4 in cells expressing wild-type or mutant SEPT12. Scale bar, 10 μm.
(PDF)

S6 Fig The presence of pre-complex SEPT7-6-2 in wild-type and SEPT12^S196E/S196E^ testis. WT and SEPT12 KI testicular lysates were immunoprecipitated using an anti-SEPT2 antibody. The expression of SEPT2, 6 and 7 was detected using anti-SEPT2, anti-SEPT6 and anti-SEPT7 antibodies, respectively.
(PDF)

S7 Fig Mimetic phosphorylated Ser198 of SEPT12 did not affect the SEPT12-SEPT12 association. (A) Co-transfection of FLAG-SEPT12 with various GFP-SEPT12 plasmids shown on the top in NT2/D1 cells; lysates were immunoprecipitated with an anti-GFP antibody (left) or reciprocally immunoprecipitated with an anti-FLAG antibody (right). The expression of FLAG- and GFP-SEPT12 was detected using anti-FLAG and anti-GFP antibodies, respectively. (B) Wild-type or mutant SEPT12 plasmids were co-transfected with FLAG-SEPT12WT into NT2/D1 cells; immunofluorescence staining showed the subcellular patterns of GFP-SEPT12 and FLAG-SEPT12 in the cells. Scale bar, 10 μm.
(PDF)

S8 Fig A phospho-Ser198 antibody that could specifically recognize phospho-Ser198 in SEPT12 was generated. (A) Dot blot analysis showed that the phospho-Ser198 antibody specifically recognizes phospho-Ser198 peptide, but not the non-phospho peptide of SEPT12. A total of 5 ng of the phospho-Ser198 peptide or non-phospho peptide per dot was adsorbed onto the nitrocellulose membrane, and the membrane was incubated with a non-phospho antibody or phospho-Ser198 antibody. Cross-reaction was observed between the phospho-Ser198 peptide and the phospho-Ser198 antibody, but not between the non-phospho peptide and the phospho-Ser198 antibody. (B) The phospho-Ser198 antibody recognized SEPT12WT, showing a basal level of phosphorylated SEPT12. In contrast, the phospho-Ser198 signal was absent in cells expressing SEPT12S198A but dramatically increased in cells expressing SEPT12S198E. These findings demonstrated that the phospho-Ser198 antibody specifically recognized SEPT12 phosphorylation at the Ser198 residue.
(PDF)

S9 Fig Multiple sequence alignment flanking the analogous Ser198 residue of SEPT12 in human septin family members. Based on amino acid sequence similarity, the septin family was divided into four subgroups: SEPT3, SEPT7, SEPT2 and SEPT6. The bracketed amino acid residues are a consensus target motif of PKA, [R/K]-X-X-[pS/T]. The amino acid sequences were analyzed using the ClustalW2 program at EMBL-EBI.
(PDF)

S10 Fig PKA regulated SEPT12WT but not SEPT12S198A-organized structure in NT2/D1 cells. (A-D) The GFP-SEPT12WT or GFP-SEPT12S198A with or without HA-PKACA2 was overexpressed in NT2/D1 cells, and cells with GFP-filament fibers (A, C) and GFP aggregates (B, D) were counted. The batch quantification bar is based on the observation of more than 500 cells. The data are represented as the means ± SEM (n = 3). *** P < 0.001.
(PDF)
