# Supplementary material for: SEPT12 phosphorylation results in loss of the septin ring/sperm annulus, defective sperm motility and poor male fertility
Source: PLoS Genet. 2017 Mar 27;13(3):e1006631. doi: 10.1371/journal.pgen.1006631 (PMC5386304; doi:10.1371/journal.pgen.1006631)

Supplementary figure 1

A

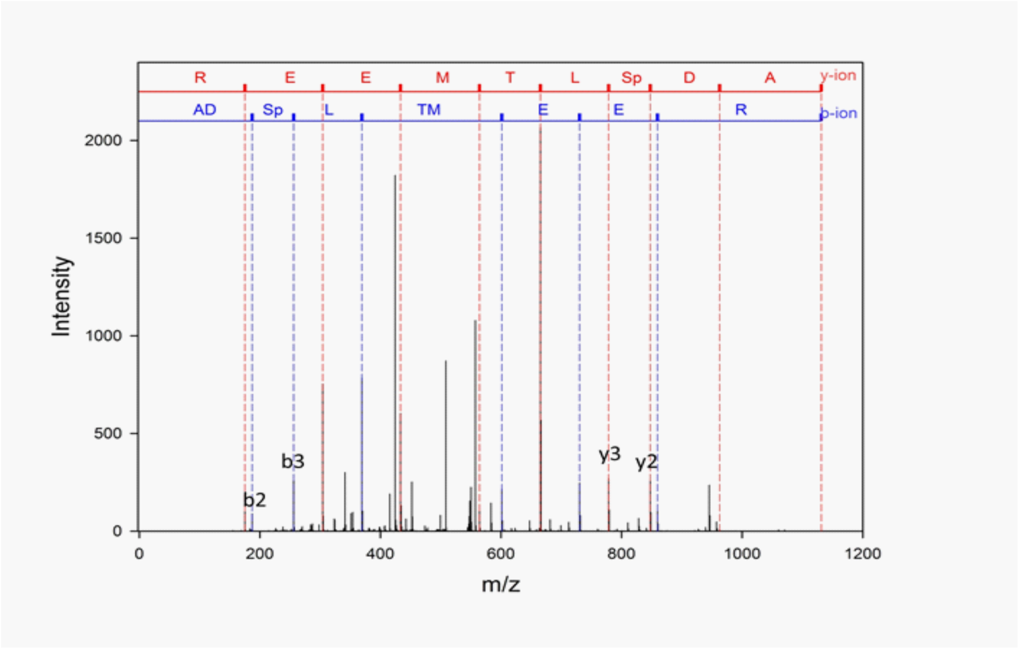

Protein Name	Accession Number	Sequence	Mascot Score	MD Score	Theoretical Mass	Mass Error	False Position Rate of Phosphorylation Site Determination Based on MD Score
SEPT12	IPI00293544	ADSpLTMEER	56.74	26.25	1130.43	0.74	0.0021
		ADSpLTMoEER	63.77	31.40	1146.43	0.67	0.0008

B

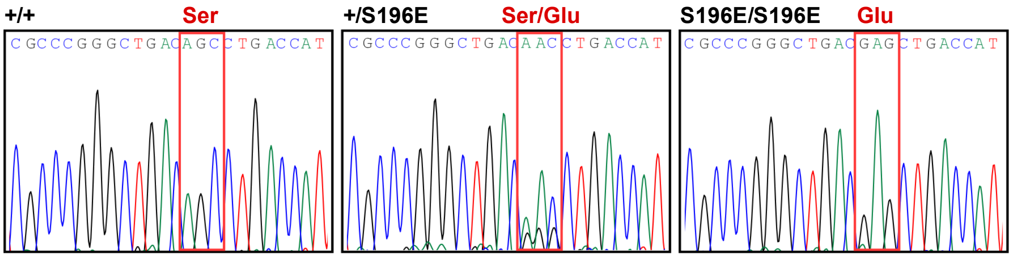

Supplement: S1 Fig — (A) Detection of human SEPT12 PTM through mass spectrometry. The MS/MS spectrum of the SEPT12 Ser198 phosphorylated peptide “196-ADSpLTMEER-204”; the y- and b-fragments for confirming Ser198 phosphorylation site are annotated. (B) Mouse genotypes were determined through DNA sequencing. Electropherograms show the genomic DNA sequence of wild-type, heterozygous and homozygous SEPT12 KI mice. Mutation from serine (Ser) to glutamate (Glu) was observed in one allele of heterozygous KI mice and in two alleles of homozygous KI mice. (PDF) [file pgen.1006631.s001.pdf]
