# Supplementary figures and images for: SEPT12 phosphorylation results in loss of the septin ring/sperm annulus, defective sperm motility and poor male fertility
Source: PLoS Genet. 2017 Mar 27;13(3):e1006631. doi: 10.1371/journal.pgen.1006631 (PMC5386304; doi:10.1371/journal.pgen.1006631)

## Supplementary figure 2

**A**

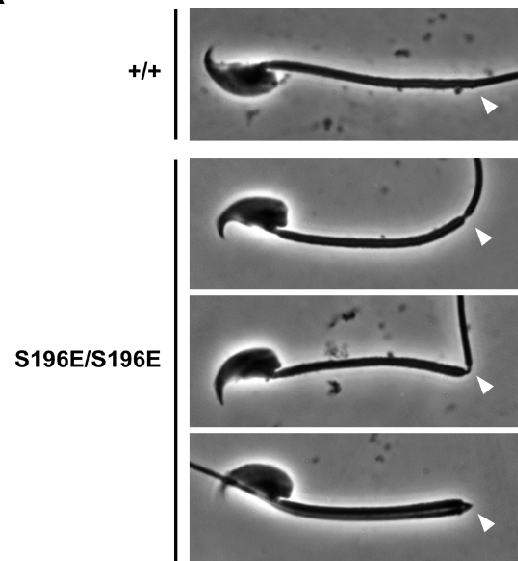

**B**

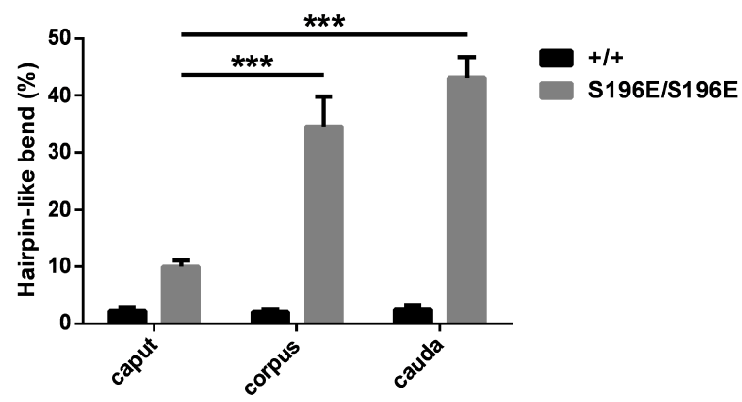

Supplement: S2 Fig — (A) SEPT12S196E/S196E sperm tails have various bending configuration. Spermatozoa were harvested form WT and SEPT12S196E/S196E cauda followed by phase-contrast microscopy analysis. The bright-field images were shown and annular regions are indicated with arrowheads. (B) Quantitative representation of the hairpin-like bend of WT and SEPT12 KI spermatozoa from epididymal caput, corpus and cauda (Each genotype, N = 4). The data are presented as the means ± SEM. ***P<0.001. (PDF) [file pgen.1006631.s002.pdf]

Supplementary figure 3

**A**

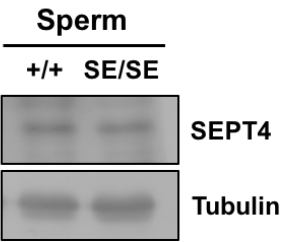

**B**

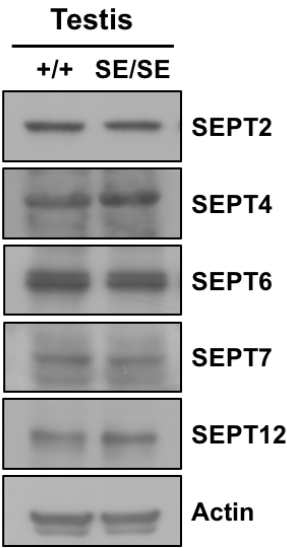

Supplement: S3 Fig — (A) SEPT4 expression in WT and SEPT12 KI spermatozoa from epididymal cauda. (B) The expression of septins including SEPT2, 4, 6, 7 and 12 in WT and SEPT12 KI testis. (PDF) [file pgen.1006631.s003.pdf]

Supplementary figure 4

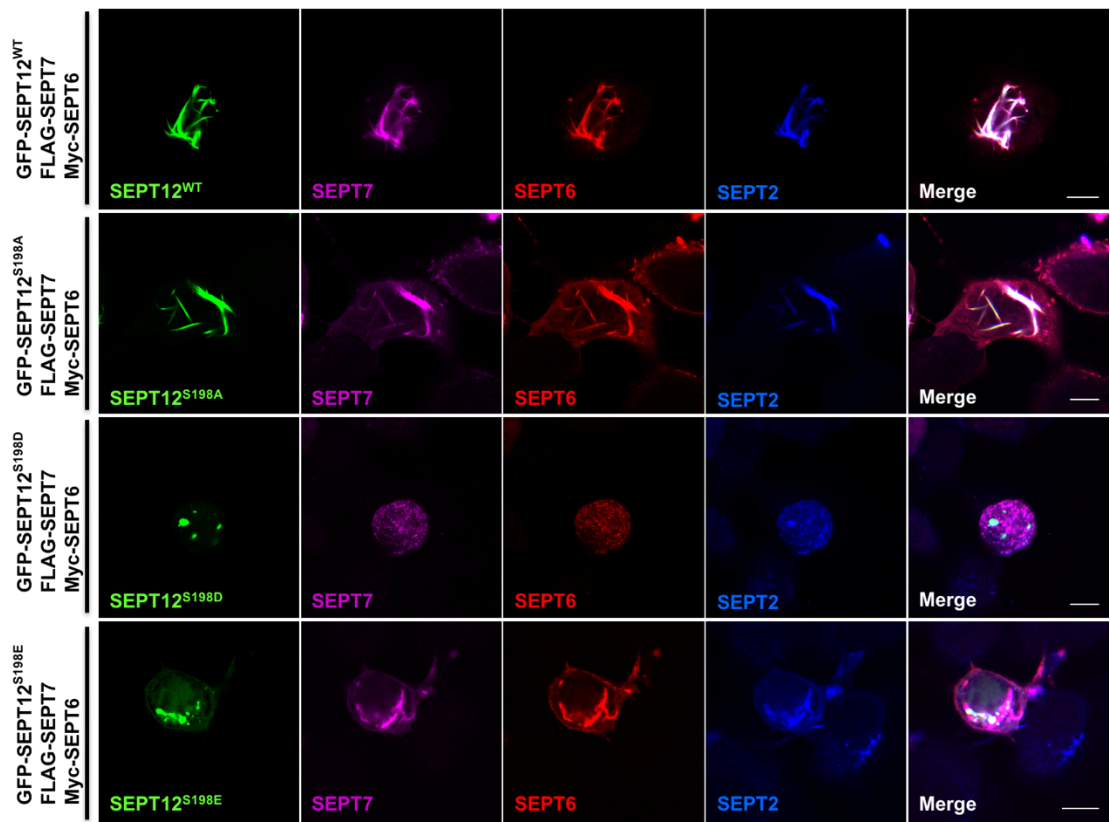

Supplement: S4 Fig — NT2/D1 cells were co-transfected with various plasmids, shown on the left. Immunofluorescence staining revealed the subcellular patterns of GFP-SEPT12, FLAG-SEPT7, Myc-SEPT6 and endogenous SEPT2 in the cells expressing wild-type or mutant SEPT12. Scale bar, 10 μm. (PDF) [file pgen.1006631.s004.pdf]

Supplementary figure 5

A

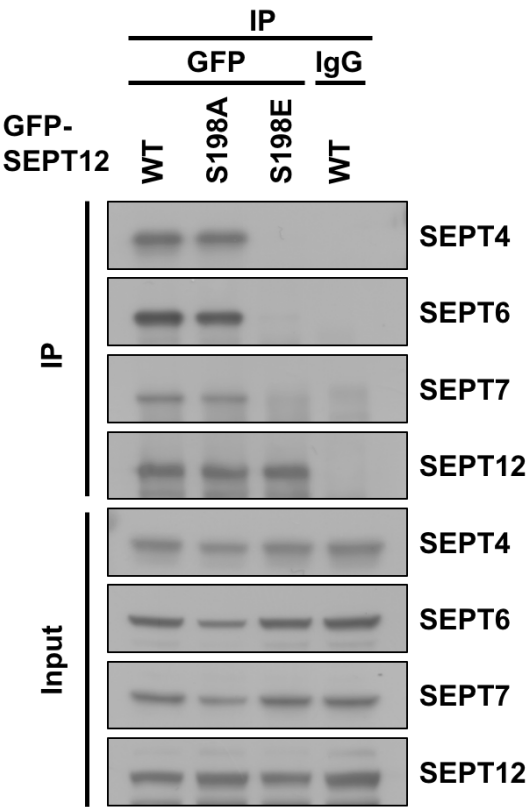

B

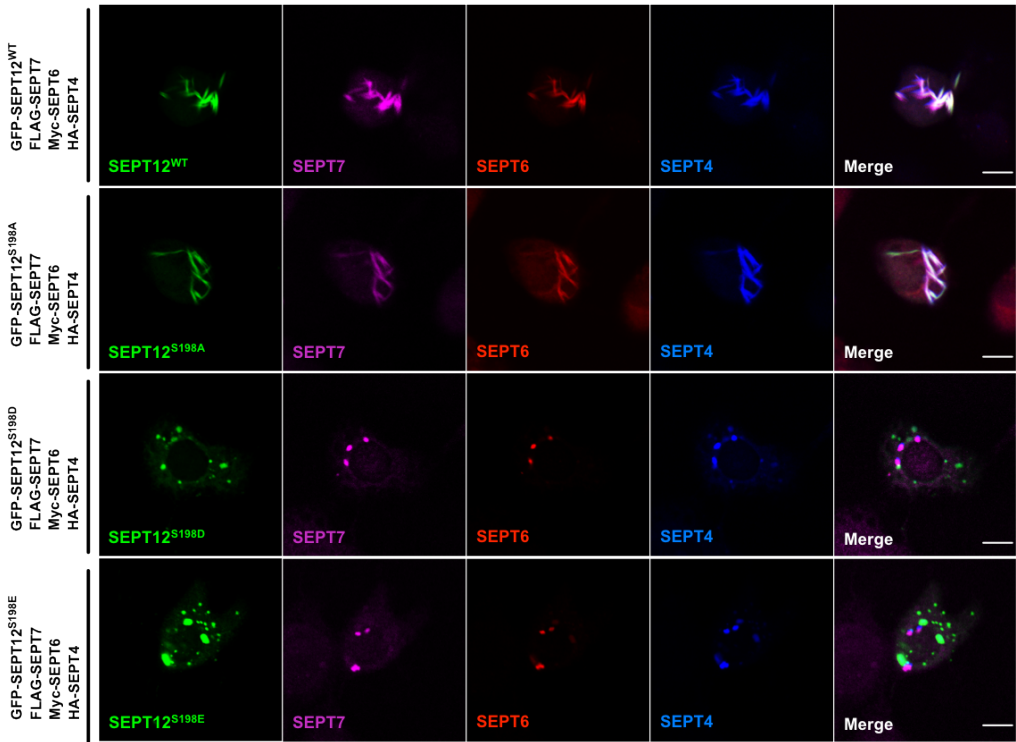

Supplement: S5 Fig — (A) Co-transfection of HA-SEPT4, Myc-SEPT6 and FLAG-SEPT7 with various GFP-SEPT12 plasmids into NT2/D1 cells; lysates were immunoprecipitated using an anti-GFP antibody. The expression of SEPT4, 6, 7 and SEPT12 was detected using anti-HA, anti-Myc, anti-FLAG and anti-GFP antibodies, respectively. (B) NT2/D1 cells were co-transfected with various plasmids, as shown on the left. Immunofluorescence staining showed the subcellular patterns of GFP-SEPT12, FLAG-SEPT7, Myc-SEPT6 and HA-SEPT4 in cells expressing wild-type or mutant SEPT12. Scale bar, 10 μm. (PDF) [file pgen.1006631.s005.pdf]

Supplementary figure 6

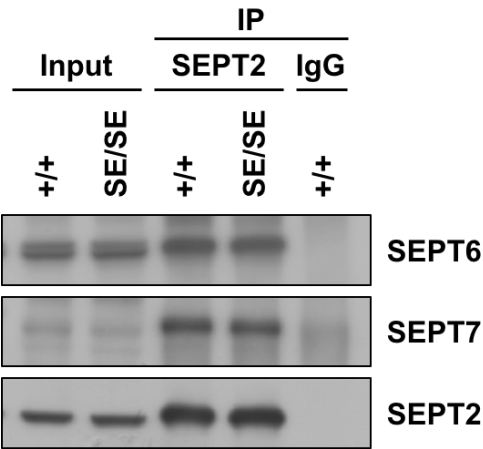

Supplement: S6 Fig — WT and SEPT12 KI testicular lysates were immunoprecipitated using an anti-SEPT2 antibody. The expression of SEPT2, 6 and 7 was detected using anti-SEPT2, anti-SEPT6 and anti-SEPT7 antibodies, respectively. (PDF) [file pgen.1006631.s006.pdf]

Supplementary figure 7

**A**

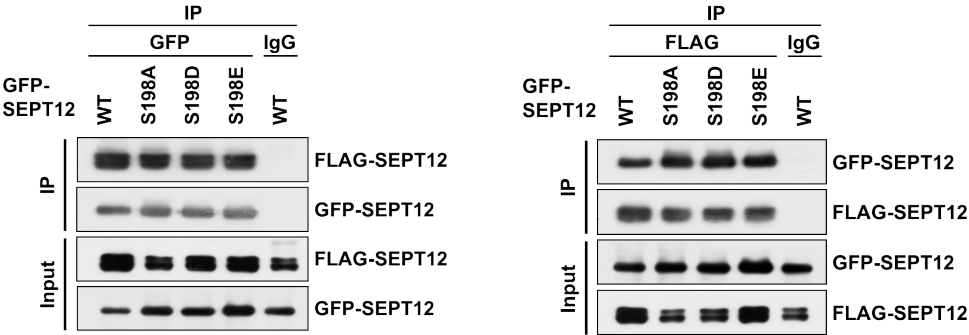

**B**

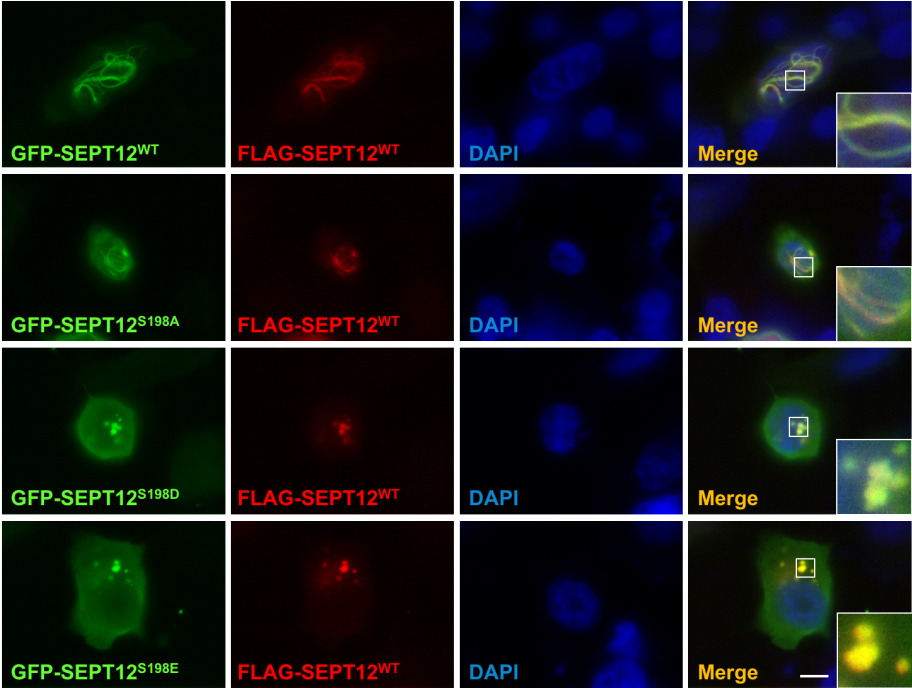

Supplement: S7 Fig — (A) Co-transfection of FLAG-SEPT12 with various GFP-SEPT12 plasmids shown on the top in NT2/D1 cells; lysates were immunoprecipitated with an anti-GFP antibody (left) or reciprocally immunoprecipitated with an anti-FLAG antibody (right). The expression of FLAG- and GFP-SEPT12 was detected using anti-FLAG and anti-GFP antibodies, respectively. (B) Wild-type or mutant SEPT12 plasmids were co-transfected with FLAG-SEPT12WT into NT2/D1 cells; immunofluorescence staining showed the subcellular patterns of GFP-SEPT12 and FLAG-SEPT12 in the cells. Scale bar, 10 μm. (PDF) [file pgen.1006631.s007.pdf]

## Supplementary figure 8

**A**

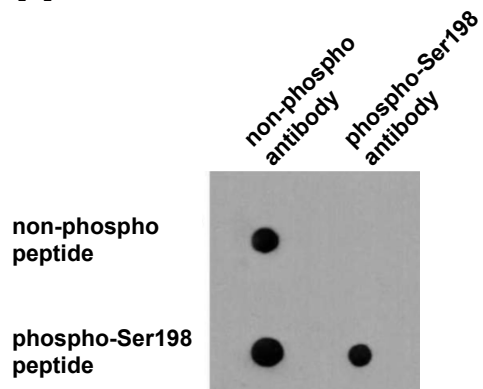

**B**

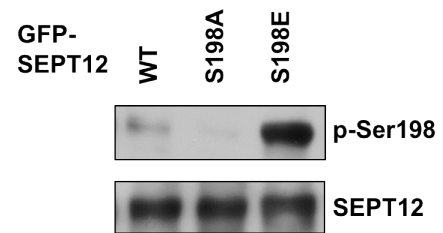

Supplement: S8 Fig — (A) Dot blot analysis showed that the phospho-Ser198 antibody specifically recognizes phospho-Ser198 peptide, but not the non-phospho peptide of SEPT12. A total of 5 ng of the phospho-Ser198 peptide or non-phospho peptide per dot was adsorbed onto the nitrocellulose membrane, and the membrane was incubated with a non-phospho antibody or phospho-Ser198 antibody. Cross-reaction was observed between the phospho-Ser198 peptide and the phospho-Ser198 antibody, but not between the non-phospho peptide and the phospho-Ser198 antibody. (B) The phospho-Ser198 antibody recognized SEPT12WT, showing a basal level of phosphorylated SEPT12. In contrast, the phospho-Ser198 signal was absent in cells expressing SEPT12S198A but dramatically increased in cells expressing SEPT12S198E. These findings demonstrated that the phospho-Ser198 antibody specifically recognized SEPT12 phosphorylation at the Ser198 residue. (PDF) [file pgen.1006631.s008.pdf]

## Supplementary figure 10

**A**

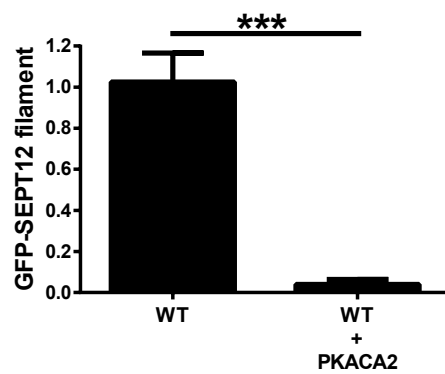

**B**

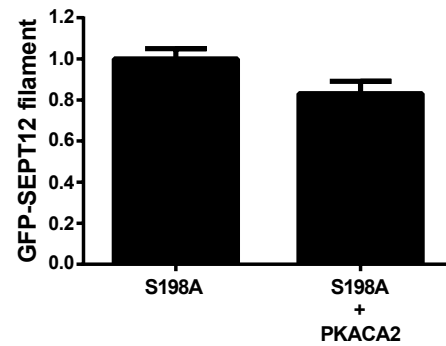

**C**

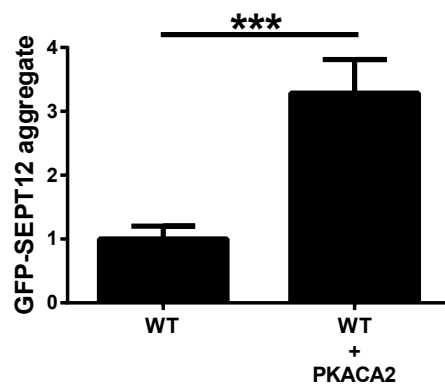

**D**

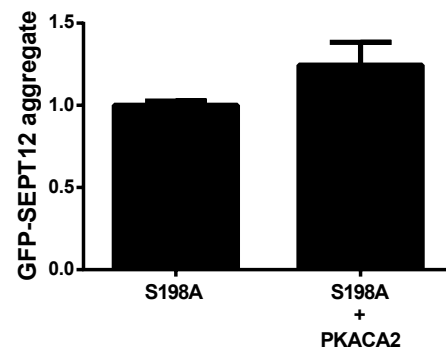

Supplement: S10 Fig — (A-D) The GFP-SEPT12WT or GFP-SEPT12S198A with or without HA-PKACA2 was overexpressed in NT2/D1 cells, and cells with GFP-filament fibers (A, C) and GFP aggregates (B, D) were counted. The batch quantification bar is based on the observation of more than 500 cells. The data are represented as the means ± SEM (n = 3). *** P < 0.001. (PDF) [file pgen.1006631.s010.pdf]
