# Supplementary material for: SEPT12 phosphorylation results in loss of the septin ring/sperm annulus, defective sperm motility and poor male fertility
Source: PLoS Genet. 2017 Mar 27;13(3):e1006631. doi: 10.1371/journal.pgen.1006631 (PMC5386304; doi:10.1371/journal.pgen.1006631)

Supplementary figure 9

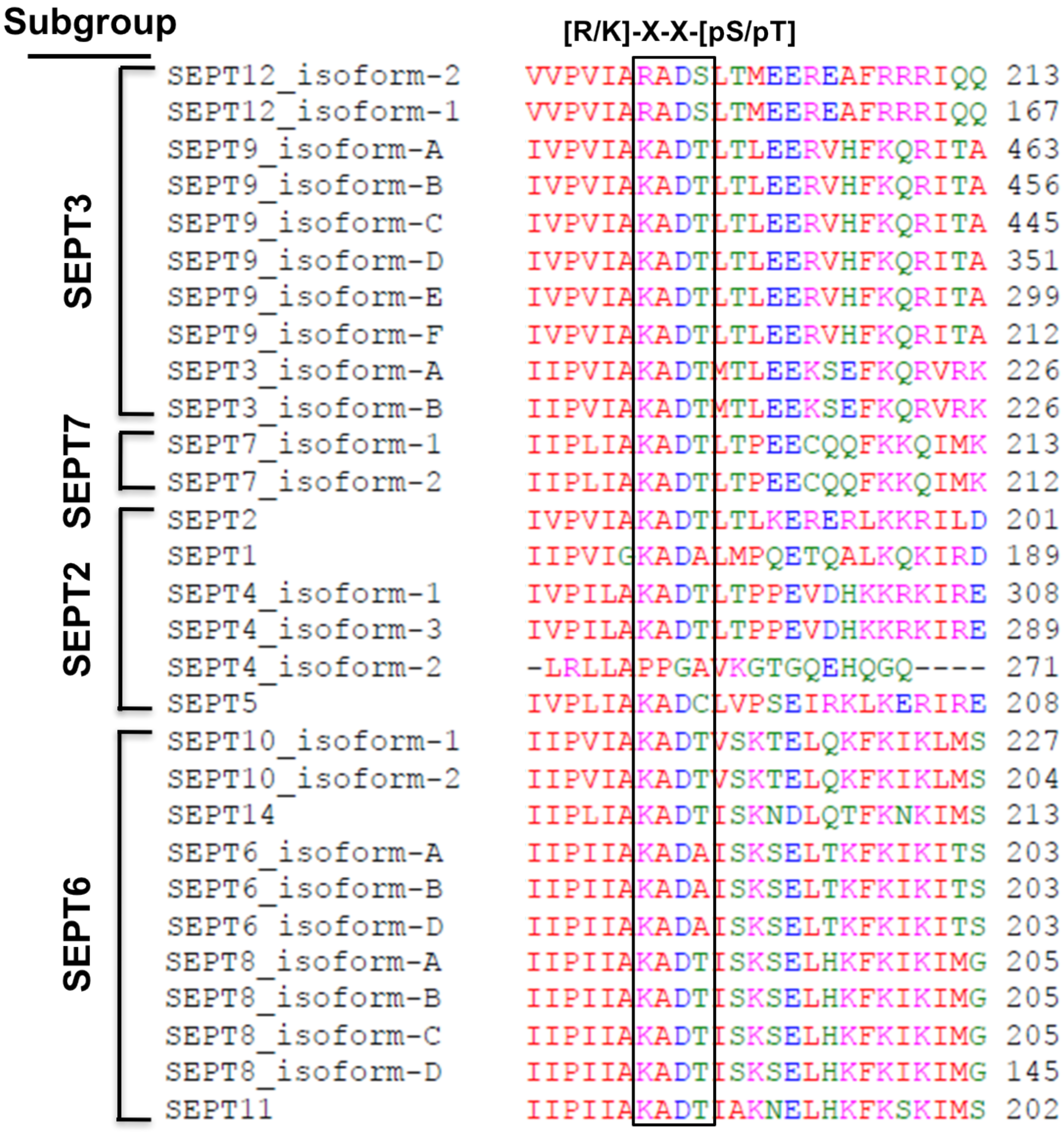

Supplement: S9 Fig — Based on amino acid sequence similarity, the septin family was divided into four subgroups: SEPT3, SEPT7, SEPT2 and SEPT6. The bracketed amino acid residues are a consensus target motif of PKA, [R/K]-X-X-[pS/T]. The amino acid sequences were analyzed using the ClustalW2 program at EMBL-EBI. (PDF) [file pgen.1006631.s009.pdf]
